# Supplementary figures and images for: Characterizing primary transcriptional responses to short term heat shock in Down syndrome
Source: PLoS One. 2024 Aug 8;19(8):e0307375. doi: 10.1371/journal.pone.0307375 (PMC11309423; doi:10.1371/journal.pone.0307375)

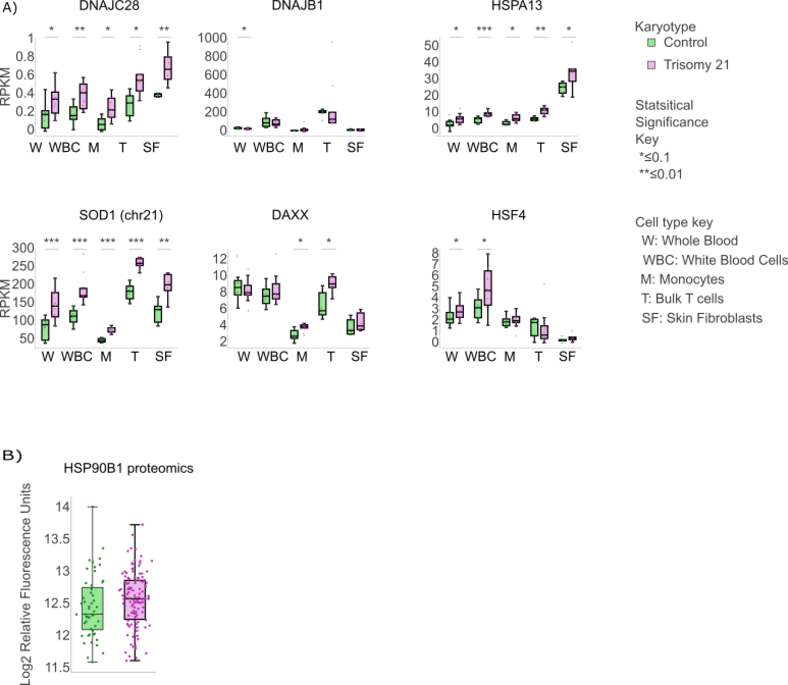

Supplement: S1 Fig — All data from the Human Trisome Project. A) Heat shock relevant genes (DNAJC28, DNAJB1, HSPA13, SOD1, DAXX, HSF4), are elevated (RNA-seq) in individuals with trisomy 21 (purple) relative to disomic controls (green). B) Proteomic data for HSP90B1 which shows increased protein levels in individuals with trisomy 21. (TIF) [file pone.0307375.s001.tif]

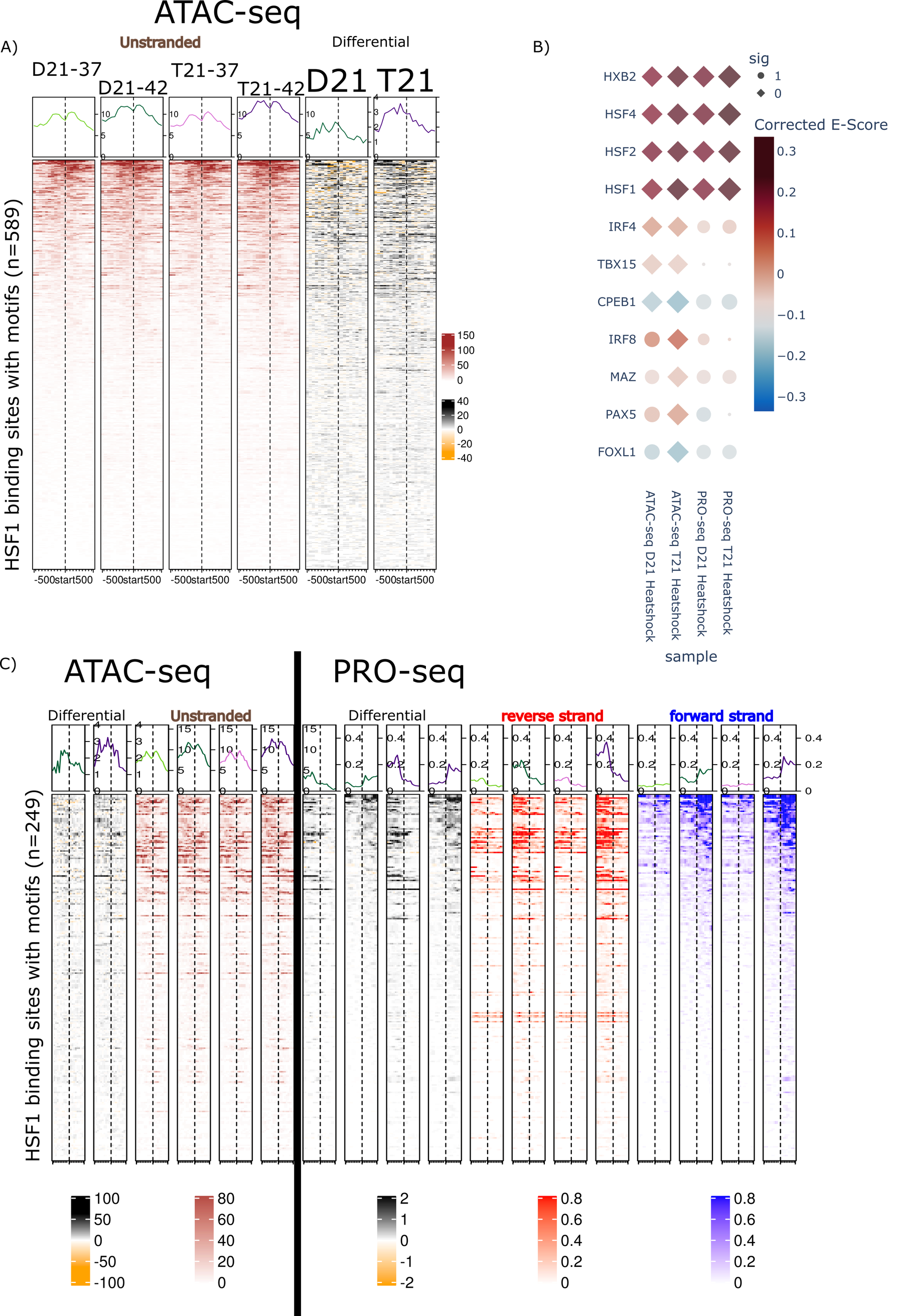

Supplement: S2 Fig — A) Heatmap of ATAC-seq data surrounding regions identified from HSF1 ChIPseq peaks with HSF1 motif instances. First four columns (in red) show DESeq2 size factor normalized ATAC-seq data at (± 500 nts) for 589 regions. The fifth and sixth columns (in black and orange) show the differential signal (heat shock—control). Top of the heat map is meta plot of the mean signal per position. One representative replicate is shown. B) A plot showing TFs reported as significantly changed by TFEA in at least one comparison. Diamonds: statically significant (p-value <1x10^−10^), Circles: not significant. Left two columns show the TFEA E-score for ATAC-seq after heat shock, whereas the right two columns show TFEA E-score for PRO-seq. E-score, or enrichment score, measures the motif co-occurrence with open chromatin (ATAC) or transcription (PRO). All TFEA analyses utilize two replicates. C) Heat maps of intergenic HSF1 bound regions (identified in ChIP-seq, requires motif) for ATAC-seq and PRO-seq data. Columns correspond to: differential ATAC-seq (black/orange, columns 1–2), ATAC-seq signal (red, columns 3–6), differential PRO-seq signal (black/orange, columns 7–10), reverse strand PRO-seq (red, columns 11–14), forward strand PRO-seq (blue, columns 16–18). Top: line graph of mean depth per position. All columns are centered at the HSF1 motif, dashed line. (TIF) [file pone.0307375.s002.tif]

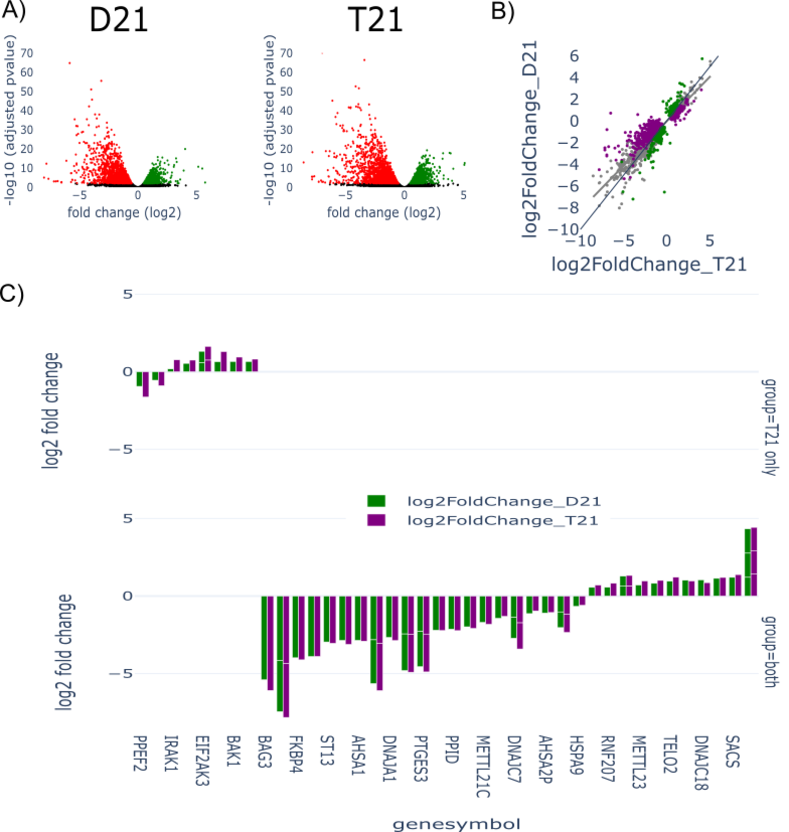

Supplement: S3 Fig — A) Volcano plots of differentially transcribed genes after heat shock. Left: Disomy, Right: Trisomy. Green: up regulated after heat shock, Red: down regulated. B) Scatter plot of log fold change observed for statistically significant differences with heat shock showing T21 (x-axis) and D21 (y-axis) samples. Colored by condition in which the significance call was made: T21 (purple), D21 (green) or both (grey). Best fit line is relative to set of grey genes. C) A bar graph of the log fold change of Heat shock genes (as defined by the GO term HEAT_SHOCK_PROTEIN_BINDING. The top plot contains the heat shock genes changed only in the trisomic sample. The bottom plot contains heat shock genes differently expressed in both cell types. (TIF) [file pone.0307375.s003.tif]

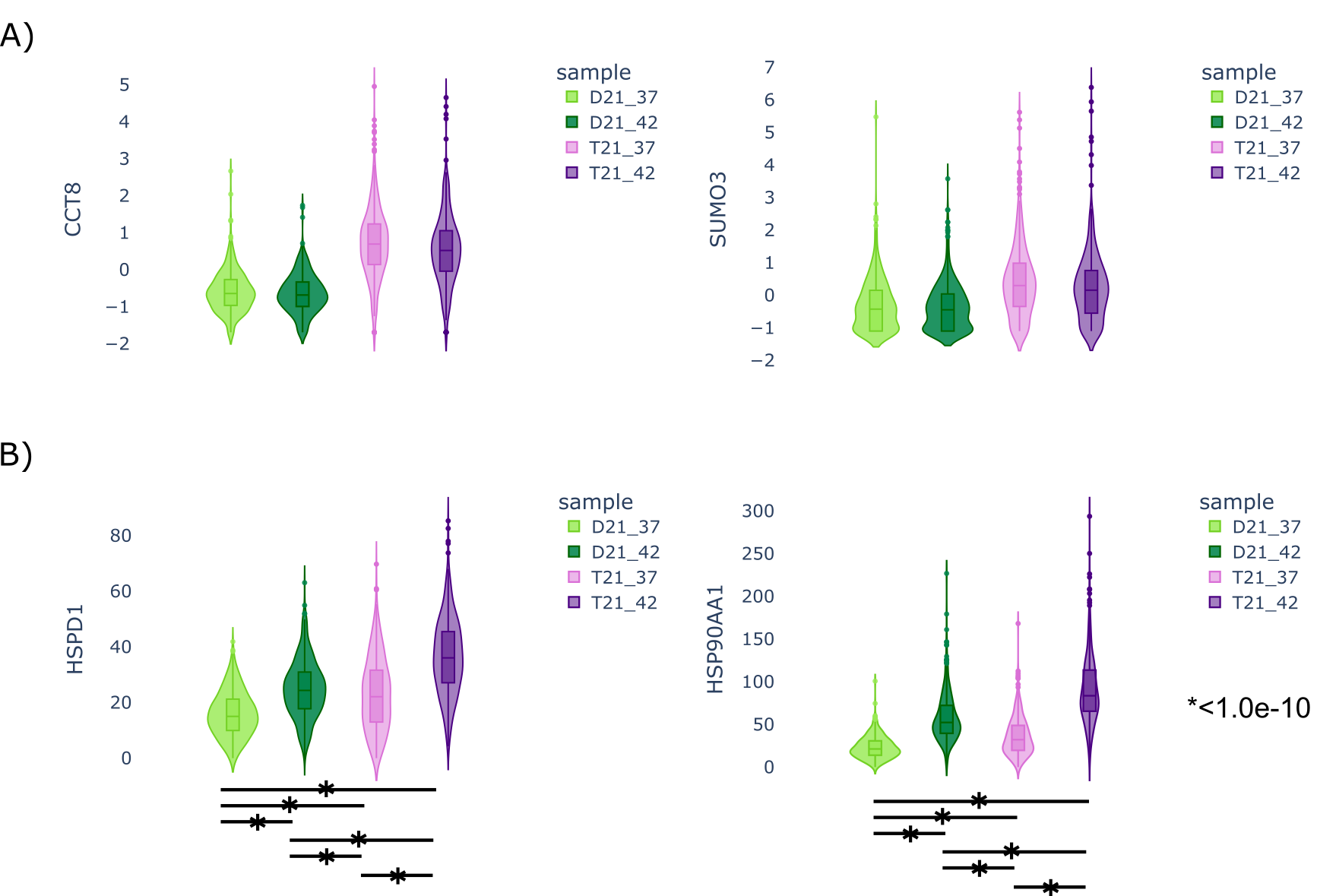

Supplement: S4 Fig — A) The same genes as in Fig 4A but with Z scores instead of raw counts. B) The same genes as in Fig 4C but with raw counts instead of Z scores. (TIF) [file pone.0307375.s004.tif]
