# Supplementary material for: Characterizing primary transcriptional responses to short term heat shock in Down syndrome
Source: PLoS One. 2024 Aug 8;19(8):e0307375. doi: 10.1371/journal.pone.0307375 (PMC11309423; doi:10.1371/journal.pone.0307375)
Supplement: Attachment — Submitted filename: plosresponsetoreviewers.pdf [file pone.0307375.s005.pdf]

Response to Reviewers

We thank the reviewers for their critical review and throughtful comments, which have led to a much improved manuscript. We have responded point-by-point to each reviewer's responses below, with some restructuring of the manuscript for flow, where appropriate. We believe these are the key points shared among reviewers:

- We agree with both reviewers that the wording throughout the paper was a bit repetitive and less than clear. We have reworded the manuscript throughout in an attempt to remove repetition and improve clarity.
- Both Reviewers were correct in noticing a lack of clarity with respect to some figure captions. We have changed to more distinct shades of green and improved the figure captions throughout.

Reviewer: 1

In this work, authors present evidences of specific heat shock signaling activation upon presence of an extra chr. 21, which is not restricted to the chr. 21 gene expression responses and rather illustrates the existence of the chronic stress phenotype. This paper adds an important peace to the puzzle, however, there are suggestion below to improve the manuscript. Mainly, the writing of the paper is not very good. It is too wordy and difficult to read. Many things are repeated again and again. The abstract should be rewritten. It is too wordy, and phrasing is not the best. Scientific English is concise and "to the point". The title of the article is too long and contains unnecessary information for the main message. I suggest changing the title.

> We thank the reviewer for his assessment. We have made extensive edits for clarity and conciseness. We have also shortened the title, as per the reviewer's suggestion.

Introduction

Lines 9-13. Very long and badly phrased sentence. It is already known (and not "unclear" as state the authors) that the responses from extra chr. 21 are not only restricted to the increased expression of genes encoded on chr. 21. The following references should be introduced PMID: 34215848, PMID: 36459979, PMID: 36496311, PMID: 27308438, PMID: 25205676

> We thank the reviewer for this suggestion. In response we have simplified these sentences and added the additional references requested. They are a great addition to this publication.

Materials and Methods

Lines 43-55 should be moved to the results. Also contains repetitive info e.g. about Nexus biobank (lines 43 and 57).

> We have moved the requested lines and removed repetition, as per the reviewer's suggestion.

Line 64. The cells were kept in waterbath at 42 degrees for 1h. So they were kept out of the incubator and lacked CO_2 for 1h? Can authors exclude the damaging effect from lack of CO_2? If there's any? And separate it from the heat shock?

This is an excellent observation from the reviewer. We now include this possibility in the text, specifically see lines 66 and 67.

Results

The figure's labeling does not correspond reality. Please revise. Lines 256-258, line 261 HSF1 levels was not "changes" and was not "not increased" but was "decreased" in certain conditions. This was also shown by Donnelly et al., 2014. Lines 272-274 Repeated sentence. Line 271, 274. You don't have to repeat all the time "1 hour".

We thank the reviewer for his observations and apologize for the lack of clarity on this figure. We have removed repetitive text and revised the labeling.

Lines 287-293 Details more suitable for Materials and Methods section. Line 317. Words are missing in the sentence. Figures 3-5. The two shades of green are very similar and difficult to distinguish on the graphs. Lines 358-360 The title is too long. P values should be indicated on the graphs e.g. of Figure 5

As suggested by the reviewer, we have moved this text to materials and methods. We have also fixed typographical errors and changed the green colors of the graphs to be more distinct. We have also added p-values to figure 5.

Reviewer: 2

In this manuscript, Cardiello et al explore heat shock-related genes from public datasets and EBV-transformed lymphoblastoid cell lines they derive from siblings with and without trisomy 21 (T21). A careful and controlled preliminary analysis is undertaken using clear bioinformatic workflows for molecular assays to identify transcriptional differences between the cell lines where T21 gene dose is a covariate that may be influencing gene expression in the context of heat-shock stress.

Assessing public available RNAseq and proteomic data on a number of tissues from Down syndrome (Human Trisome Project), early gene expression with PROseq and ATACseq for chromatin accessibility, and scRNAseq approaches comparing the lymphoblastoid cell lines, the authors conclude: 1. a number of Heat shock genes are inconsistently upregulated in T21 cohorts compared to disomic controls, including HSF1, a heat shock regulating transcription factor, from publicly available datasets 2. comparison between the lymphblastoid cell lines (T21 and disomic control) show - increased chromatin accessibility at specific heat shock response elements (HSF1, SERPINH1) with heat shock stress with an inferred increased in TF activity using Transcription Factor Enrichment Analysis of ATACseq data, and further (and perhaps difficult to interpret) analysis undertaken for sites enriched for one HSP TF, HSF1 binding by ChIPseq (Fig S3) 3. Heat shock response was associated with greater deferentially transcribed genes (up and down) in T21 cell line with enrichment in heat shock associated TFs (HSF1, HSF2, HSF4, HXB2), with PRO-seq suggesting "more robust" nascent RNA transcription in T21 cell line with heat shock. 4. Clonal heterogeneity in the cell lines with heat shock using scRNAseq was examined, demonstrating that at least for some certain heat shock responsive genes, changes in transcription appear at some degree to be at a "population" level, rather than dramatically responding outlier clones.

The authors appropriately state that further studies are required to understand the implications of these preliminary, albeit convincing, observations in blood and particularly, immune cells.

Major comments

1. Much of the detailed molecular characterisation of heat shock response is conducted on disomic and T21 lymphoblastoid cell lines from (I assume) genetically related brothers that had been immortalised by identical batch EBV transduction. Could the authors comment about the generalisability of their observations for these "single" replicate analyses. Are there other available sib pairs (disomic and trisomic) for instance in the Nexus biobank to be able to undertake replicate analysis of at least some of the assays? If not, is there benefit/possibility of deriving and undertaking a subset of analyses (eg. RNAseq) from transduction of the blood cells with another EBV transduction (as pseudo-biological replicates)? If not, I think these observations need to be qualified in this context by the authors.

> We agree the results of this publication must be confirmed in other cell lines from other individuals. In the absence of other cell lines, a new transfection of these same individuals would be useful. However, as the funding for this project has ended, we cannot complete additional samples or replicates.
>
> In light of this accurate comment, though, we have brought up this criticism in the paper's discussion section. We agree with the review in believing more work is needed to confirm this result generally applies to all individuals with Down syndrome.

2. While perhaps inconsistently described in the methods, replicates undertaken for each assay, particularly using the lymphoblastoid cell lines, should be described in the legend.

> We have added the information about the replicate numbers to the figure legends.

3. The specific public dataset data used for analysis from the Human Trisome Project, in this manuscript should be provided (eg. which coded sample identifiers for each sample) as a supplementary table for reproducibility.

> We apologize for the lack of clarity on this point. The data from the Human Trisome Project was found on the website http://explorer.trisome.org/transcriptome/ and therefore we do not have sample ids. However, the reviewer's point made us realize that we did an inadequate job in describing how this data was obtained, and therefore we have added a paragraph in the methods section describing how these graphs were produced.

4. Fig 3F is missing a legend

> We thank the reviewer for catching this mistake. It has been corrected.

5. Supp Fig 3C: Differential ATACseq and PROSeq data requires some clarity with regard to the visual plots, including the directionality of the data (the conditions appear to be missing for each column (cf Fig S3A) and what each column represents (?technical replicates I am assuming - see comment 2)

> We apologize for the lack of clarity in our description of Figure S3. We have added more information to the description. "Differential ATACseq and PROSeq" is the 42-degree plots minus the 37-degree plots. Therefore, the differential shows how much higher the signal at each site was in the heat shock. The bottom heat map section shows each of the individual genomic locations on the y-Axis. In Figure S3A 589 genomic locations are shown and in Figure S3C there are 249 genomic locations. There are more sites in Figure S3A because all sites with any ATAC signal are shown whereas Figure S3C contains only HSF1 bound sites that do not overlap genes. We removed sites that overlap genes in Figure S3C becuase PRO-seq for sites with genes would include both transcription of the gene and transcription of the HSF1 site via eRNA. We have clarified the caption accordingly.

6. missing "to" in "next sought (to) confirm" line 306 page 11

> We thank the reviewer for catching this mistake. It has been corrected.
